# Endovascular management of acute and subacute venous thoracic outlet syndrome

**DOI:** 10.3389/fsurg.2024.1302568

**Published:** 2024-02-19

**Authors:** Mark G. Davies, Joseph P. Hart

**Affiliations:** ^1^ Department of Cardiovascular Outcomes, Center for Quality, Effectiveness, and Outcomes in Cardiovascular Diseases, Houston, TX, United States; ^2^ Division of Vascular and Endovascular Surgery, Medical College of Wisconsin, Milwaukee, WI, United States

**Keywords:** venous thoracic outlet syndrome, endovascular therapy, outcomes, DVT, pharmaco-mechanical thrombolysis, subclavian vein stenosis, central venous stenosis, subclavian venoplasty

## Abstract

Approximately 3% of all patients presenting with Thoracic Outlet Syndrome have a venous etiology (vTOS), which is considered “effort thrombosis”. These patients will present with symptomatic deep venous thrombosis or focal subclavian vein (SCV) stenosis. Endovascular management of vTOS occurs in several phases: diagnostic, preoperative therapeutic intervention before decompression, postoperative interventions after decompression, and delayed interventions in the follow-up after decompression. In the diagnostic phase, dynamic SCV venography can establish functional vTOS. Approximately 4,000 patients have been treated for vTOS and reported in the literature since 1970. Declotting of the SCV was followed by surgical decompression in 53% of patients, while in the remainder, surgical decompression alone (18%), endovascular intervention alone (15%), or conservative therapy with anticoagulation (15%) was performed. The initial intervention was predominantly catheter-directed thrombolysis, with <10% of cases undergoing concomitant balloon angioplasty. 93% of cases were successful. In the postoperative phase, balloon angioplasty was performed to correct residual intrinsic SCV disease after vTOS decompression in under 15% of cases. Stents were rarely deployed. Symptom relief was reported as 94 ± 12% (mean ± SD) and 90 ± 23%, respectively for declotting with decompression and declotting alone. In the delayed phase, balloon angioplasty was performed in under 15% of cases to re-establish patency.

## Introduction

Venous Thoracic outlet syndrome (vTOS) is characterized by compression and often thrombosis of the subclavian vein. The development of a DVT in the subclavian associated with extrinsic compression is commonly referred to as “effort thrombosis” or “Paget-Schroetter syndrome” (1). The proportion of all Thoracic Outlet Syndrome that is considered venous in etiology is estimated to be approximately 3% of all reported cases (2). Most patients presenting with vTOS are males in their second or third decades, but the condition can be found in older adults (3). The acuity of presentation in vTOS is based on the patient's presenting arm symptoms of swelling, pain, and distended veins (4). The Society for Vascular Surgery reporting standards have described “acute” vTOS as that of a patient presenting with symptoms within the first 14 days of symptoms. Patients with “subacute” vTOS were defined as presenting with symptoms or signs from 14 days to 3 months, while patients with subclavian vein disease that have been documented for 3 months or more are described as “chronic” (5). The aim of this review is to examine the current state of endovascular interventions in comprehensive therapy for vTOS.

### Anatomy

The subclavian vein (SCV) arises from the confluence of the cephalic vein with the axillary vein and courses into the upper mediastinum to the confluence of the jugular vein to form the brachiocephalic vein. The SCV traverses a short and narrow musculoskeletal path between the clavicle and 1st rib, termed the costoclavicular junction (CCJ), resulting in a confined domain that can lead to both physiological and pathological compression of the vein (6). This narrowed CCJ space leads to physiological compression with arm movements, functional compression without symptoms, and symptomatic compression leading to upper extremity symptoms (4).

### Pathophysiology

The pathophysiology of vTOS is a combination of extrinsic mechanical compression of the SCV at the CCJ, intrinsic venous mural injury to the vein due to the extrinsic compression and ongoing shoulder girdle movement, and eventually luminal venous thrombosis on the surface of the subclavian vein (7). These pathophysiological processes lead to either an acute deep venous thrombosis (DVT) or a significant often dynamic focal venous stenosis (7, 8). An acute SCV DVT or stenosis will induce venous hypertension in the ipsilateral upper extremity and tissue edema which leads to symptoms of pain, swelling decreased range of movement, and visible superficial collateral veins on the chest and upper arm. Without intervention, superficial and deep collateral veins will develop and the normal healing process of a deep venous thrombosis within the SCV should occur. These may or may not abate the patient symptoms.

### Modalities

#### Thrombolysis

Thrombolytic or fibrinolytic agents are systemically administered exogenous plasminogen activators whose purpose is the breakup of the acute clot. These compounds are serine proteases that cleave native plasminogen into active plasmin within the body. Plasmin, also a serine protease, cleaves fibrin into various fibrin degradation products, thus destabilizing the structure of a fresh clot (fibrin matrix with platelets) and thus allowing its dissolution. The most frequently used thrombolytic agents reported are: S*treptokinase, Anistreplase*, U*rokinase*, A*ctivase*, R*eteplase* and *Tenecteplase*. Plasminogen activators may be administered intravenously through site non-specific, site-specific, or enhanced site-specific modalities over 1–72 h:

• *Systemic thrombolysis:* A dose of a plasminogen activator (2–10 mg tPA) is administered through a peripheral intravenous catheter upstream of the clot burden over 1 h. This technique has fallen into disuse with the advent of catheter based therapies for peripheral and central DVTs (9).
• *Catheter-directed thrombolysis:* Catheter-directed thrombolysis (CDT) is a combination of selective catheter placement and infusion of thrombolytic agents. A multi-side-hole catheter is placed within the clot, a continuous infusion of a plasminogen activator is administered into the clot at 0.5–1.0 mg/hr of tPA over 12–72 h using an intravenous pump (9–12).
• *Ultrasound-enhanced thrombolysis:* After the clot is traversed with a multi-side-hole catheter that has ultrasound emitters present, an infusion of a low-dose plasminogen activator is administered into the clot with concomitant ultrasound activation over 12–72 h at 0.5 to 1 mg/hr for tPA (9, 13). The penetration of the drug and clot dissolution are mediated by stable cavitation or sustained bubble activity induced by the ultrasound frequency.
• *Pulse spray thrombolysis:* Following the placement of either a multiple-side hole catheter or a rheolytic catheter into the body of the clot, a highly concentrated fibrinolytic agent (5–15 mg of tPA in 100 mls normal saline) is injected directly into the thrombus as a brief high-pressure spray to facilitate drug penetration and pneumatically disrupt the clot. The lytic agent is administered over 15–30 min (9, 14, 15).

#### Rheolytic catheters

Rheolytic catheters are delivered percutaneously to the site of the clot and use a mechanical distribution technique which can be a rotating catheter extension (e.g., Trellis™, *Covidien*) or a high-pressure, high-speed fluid jet (e.g., Angiojet™, *Boston Scientific*) to mechanically disrupt the clot in a focal area and retrieve it (16, 17). Several devices have additional enhancements to isolate the area of the disruption (e.g., proximal and distal balloons) or to prevent distal embolization of the morselized fragments of the clot (e.g., a distal embolic filter). These devices effectively disrupt and remove mature and resistant clots that traditional catheter-directed thrombolysis fails to lyse effectively. The combination of a rheolytic catheter and thrombolysis is referred to as pharmaco-mechanical thrombolysis (PMT).

#### Percutaneous thrombectomy catheters

Percutaneous thrombectomy catheters are delivered percutaneously to the site of the clot and, in a thrombolytic independent manner, disrupt and retrieve blood clots by capturing the clot in a wire mesh basket system or directly aspirating out the clot. These systems come in both large-bore and small-bore designs (13, 17).

#### Percutaneous embolectomy catheters

Percutaneous embolectomy catheters (e.g., over the wire or plain Fogarty catheters™, *Edwards Life Sciences*) are delivered percutaneously over the wire to the site of the clot, and the catheter balloon is inflated at the most distal end of the clot from the sheath. With the embolectomy balloon inflated, the clot is captured and withdrawn to the area of the initial sheath access, where a large bore sheath is under direct suction to facilitate extraction of the displaced clot material (17, 18).

#### Balloon angioplasty and stenting

Angioplasty balloons and stents are delivered percutaneously over the wire to the site of the clot, In general, with acute thrombus, thrombolysis or percutaneous thrombectomy will have been performed to remove acute friable clot. In vessels with chronic obstructive thrombo-fibrotic lesions, no pre-emptive declotting procedures are required (19). Balloon angioplasty can be employed with plain balloons, wall-modifying balloons (cutting or scoring), or high-pressure balloons (20). If greater than 30% residual stenosis remains, bare metal or covered stents can be deployed to increase luminal diameter (19).

### Endovascular management of vTOS

Endovascular management for vTOS consists of a set of diagnostic and therapeutic interventions that can stand alone or be temporizing bridges before a durable intervention such as CCJ decompression is performed (21). These endovascular interventions occur in several phases: diagnostic, preoperative therapeutic intervention before decompression, postoperative therapeutic interventions after decompression, and delayed therapeutic interventions in the follow-up after decompression.

#### Diagnostic phase

In the diagnostic phase, dynamic venography can easily establish functional vTOS (22). The venography is able to assess patency and physiological changes when the arm is placed in positions that induce subclavian vein stenosis at the costoclavicular junction (23). While venography is a 2-dimensional testing modality, the increasing use of 3-dimensional intravascular ultrasound can offer information on the status of the vessel and the compression point in the outlet in three dimensions (24, 25).

#### Preoperative therapeutic phase

In the therapeutic phase where thrombosis is identified, catheter-directed thrombolysis, pharmaco-mechanical thrombolysis, or mechanical thrombectomy are performed to clear the thrombosis burden (26, 27). In the acute phase, 0–14 days, thrombectomy and lysis can be used with equal clot reduction but different treatment intervals. In the subacute phase, thrombolysis rather than thrombectomy is more common to remove any fresh clot and allow for angioplasty and/or stenting. “The primary endpoint for these therapies was the resolution of >70% of thrombus. The immediate overall technical success rate for acute thrombus removal described in the literature is over 90% in CDT, 100% in PMT, and 100% for percutaneous thrombectomy systems (19). Technical success is associated with an equivalent rate of symptom relief. An initial subclavian vein balloon angioplasty may be performed to optimize flow at the conclusion of the declotting procedure (28). However, given that the luminal narrowing is primarily due to extrinsic compression at the CCJ, balloon angioplasty rarely results in complete resolution of the venous stenosis. More robust balloon angioplasty interventions carry the risk of rupture, intimal disruption with recurrent thrombosis, or perivascular inflammatory response, which may complicate any planned open decompressive procedure (4, 29). Initial declotting followed by prompt first rib resection within 2 weeks of initial thrombosis demonstrates a greater than 90% success rate with significant symptom relief and return of full function in the upper extremity (19). In a systematic review that yielded 6 appropriate articles, early decompression surgery within two weeks after percutaneous declotting procedures appears safe and effective (30). However, if decompression is performed after 6 weeks of symptom onset and the initial declotting, the success rate is reduced to <60%. Most of the patients with a delayed presentation (>6 weeks of symptom onset) will still benefit from a decompression accompanied by a venous reconstruction if surgically fit for a procedure (4).

#### Advanced therapeutic modalities

For patients who present with a chronic venous occlusion, it may not be possible to traverse the subclavian venous lesion percutaneously and recanalize the vein. While most patients will be considered surgically fit enough to progress to a venous repair with a patch, an autologous or allograft bypass, or an ipsilateral jugular vein turndown, new recanalization techniques have been described that combine advanced imaging systems and sharp recanalization with a needle or radiofrequency power wire to allow traversal of the chronic lesion (31, 32). The vein can then be stented with a covered stent. The majority of these cases have been performed in patients with vTOS, and thus the use of these modalities remains unclear.

#### Post-operative therapeutic phase

In the postoperative therapeutic phase, when decompression has been successfully achieved, venography coupled with angioplasty and stenting can be performed to correct any persistent intrinsic venous disease that results in stenosis. This venography is often performed prior to closure of the decompression surgical incisions to allow for possible open venous thrombectomy, open surgical patching/repair of the subclavian vein, a jugular vein turndown to the axillary vein, or a bypass of the subclavian vein from the axillary vein to the jugular vein (33). The endovascular management of these lesions has been to employ balloon angioplasty and selective stenting with an open cell or a covered stent.

#### Delayed therapeutic phase

In the delayed phase, venography and endovascular intervention is performed if noninvasive imaging detects recurrent venous stenosis or ipsilateral upper extremity symptoms recur. Most studies have shown that decompression surgery is associated with a significantly lower rate of imaging-detected signs of persisting vascular compression (34). However, the rate of persisting clinical symptoms is comparable to those treated only by endovascular or conservative therapy.

### Complications of endovascular interventions

#### Thrombolysis

The major disadvantage of systemic thrombolysis is the increased risk of serious bleeding complications, with intracranial hemorrhage carrying the highest mortality. Intravenous tPA carries a 3%–6% risk of intracranial hemorrhage. Early thrombolytics were also associated with allergic reactions, which are now relatively rare. The main complication of CDT remains bleeding. However, in the case of CDT, significant bleeds are usually confined to the site of venous puncture, and intracranial bleeding is rare.

#### Rheolytic catheter

The major complication of these catheters is hemolysis and the release of of free hemoglobin that can result in acute kidney injury due to the toxic effects of free hemoglobin. The combination of a rheolytic therapy with a thrombolytic agent is associated with a bleeding risk.

#### Percutaneous thrombectomy catheters

The major complications of percutaneous thrombectomy are vessel wall injury, proximal embolization, blood loss, and associated vessel wall injury.

#### Percutaneous embolectomy catheters

The major complications of percutaneous embolectomy are proximal embolization, failure to achieve clot extraction, blood loss, vessel wall injury, and associated vessel wall injury.

#### Balloon angioplasty and stenting

The major complication of balloon angioplasty is failure to achieve luminal gain, perforation, and immediate and early thrombosis. The major complication of stent placement is compression of the stent, dislodgement of the stent, fracture of the stent at the level of the first rib, and immediate and early thrombosis.

### Current practice

**Figure 1:**
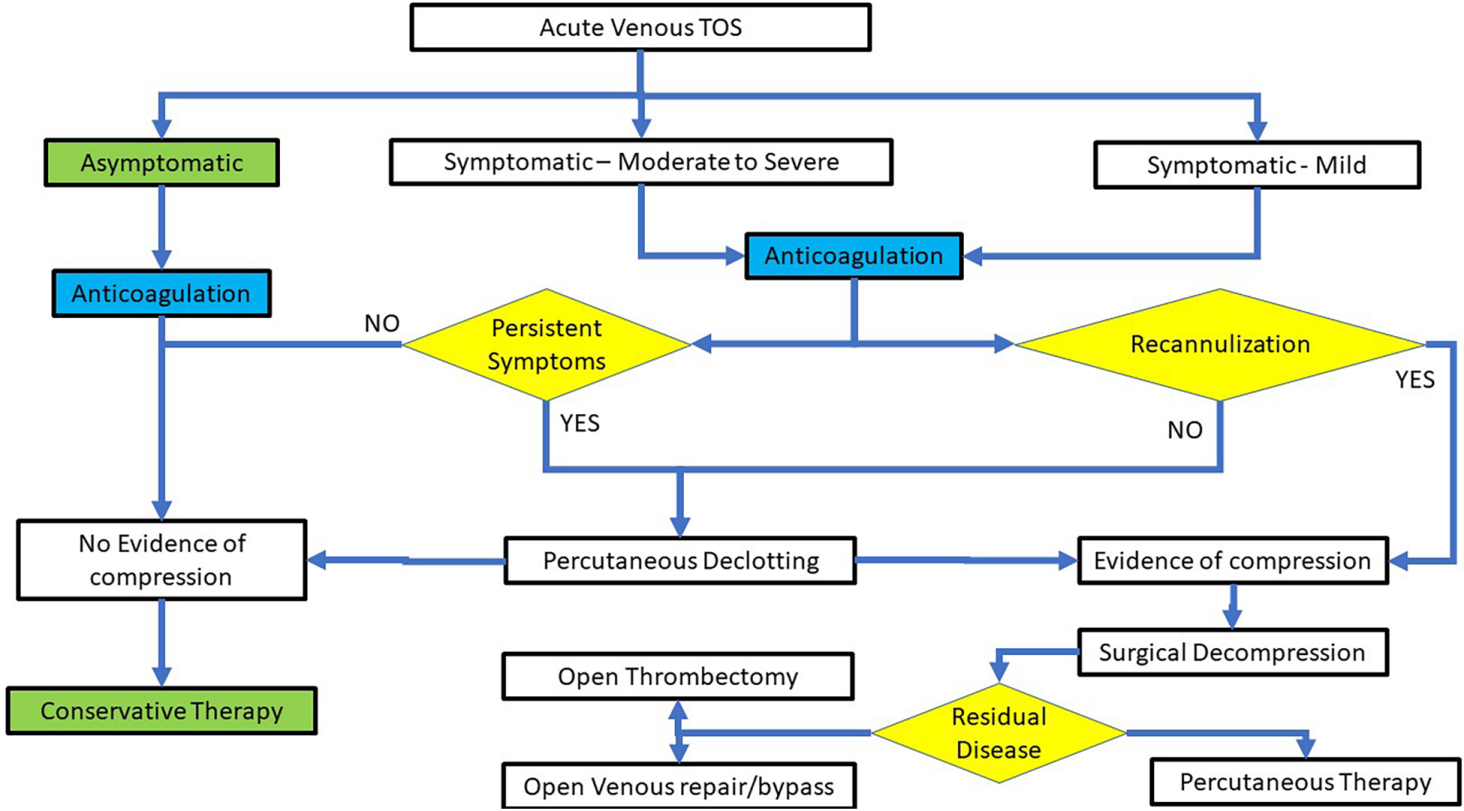
A treatment algorithm for the endovascular therapy of acute VTOS.

While there are no randomized controlled trials that can inform on the optimal therapeutic strategy for vTOS, a recent systematic review and meta-analysis strongly suggests that higher rates of thrombus removal and symptoms resolution is achievable with SCV thrombolysis, followed by first rib resection (19, 34). Current clinical practice dictates that with the diagnosis of deep venous thrombosis (DVT) in the subclavian vein, anticoagulation therapy is commenced to minimize proximal and distal clot progression before consideration of the diagnosis and definitive management of vTOS (34). At present, the commonest pattern of care for acute vTOS presenting with a DVT consists of ipsilateral contrast venography for diagnostic verification followed by a declotting procedure (catheter-directed thrombolysis, pharmaco- mechanical thrombectomy or mechanical thrombectomy) to debulk the subclavian vein clot, restore patency. Once the lumen is re-established, there is a need to define the presence or absence of intrinsic or extrinsic stenosis at the CCJ. Patients with a diagnosis of CCJ stenosis (i.e., vTOS) are frequently referred for a decompressive procedure within 6 weeks, but alternatively, they are offered a standard duration of anticoagulation therapy recommended by current guidelines for a peripheral DVT (35). In those that are treated endovascularly and need decompression, a short interval (2–6 weeks) is recommended prior to surgical decompression of the CCJ by first rib resection (30). This is achieved through various open surgical techniques (36). Immediate venography after surgical decompression is then performed and, when appropriate, is followed by percutaneous intervention or open reconstruction of the subclavian vein. A potential treatment algorithm for vTOS is shown in Figure 1.

## Conclusion

Diagnostic and therapeutic endovascular interventions for vTOS are a necessary part of managing the disease. Removal of the clot using any modality is associated with rapid symptomatic benefit with or without subsequent surgical decompression. Once treated, the recurrence rate and the need for secondary interventions remain low.

## References

[1] DiLosa KL Humphries MD. Epidemiology of thoracic outlet syndrome. Semin Vasc Surg. (2021) 34(1):65–70. 10.1053/j.semvascsurg.2021.02.008 33757638

[2] Illig KA Rodriguez-Zoppi E Bland T Muftah M Jospitre E. The incidence of thoracic outlet syndrome. Ann Vasc Surg. (2021) 70:263–72. 10.1016/j.avsg.2020.07.029 32771464

[3] Ferrante MA. The thoracic outlet syndromes. Muscle Nerve. (2012) 45(6):780–95. 10.1002/mus.23235 22581530

[4] Illig KA Doyle AJ. A comprehensive review of paget-schroetter syndrome. J Vasc Surg. (2010) 51(6):1538–47. 10.1016/j.jvs.2009.12.022 20304578

[5] Illig KA Donahue D Duncan A Freischlag J Gelabert H Johansen K Reporting standards of the society for vascular surgery for thoracic outlet syndrome. J Vasc Surg. (2016) 64(3):e23–35. 10.1016/j.jvs.2016.04.039 27565607

[6] Urschel HC Pool JM Patel AN. Anatomy and pathophysiology of VTOS. In: Illig K Thompson R Freischlag J Donahue D Jordan S Edgelow P, editors. Thoracic Outlet Syndrome. London: Springer (2013). p. 339–43. 10.1007/978-1-4471-4366-6_49

[7] Audu CO Vemuri C Urschel HC Pool JM Patel AN. Anatomy and pathophysiology of venous thoracic outlet syndrome. In: Illig KA Thompson RW Freischlag JA Donahue DM Jordan SE Lum YW, editors. Thoracic Outlet Syndrome. Cham: Springer (2021). p. 487–94. 10.1007/978-3-030-55073-8_53

[8] Navarrete S Solar C Tapia R Pereira J Fuentes E Palomo I. Pathophysiology of deep vein thrombosis. Clin Exp Med. (2023) 23:645–54. 10.1007/s10238-022-00829-w 35471714

[9] Izcovich A Criniti JM Popoff F Lu L Wu J Ageno W Thrombolytics for venous thromboembolic events: a systematic review with meta-analysis. Blood Adv. (2020) 4(7):1539–53. 10.1182/bloodadvances.2020001513 32289164 PMC7160254

[10] Vedantham S Sista AK. How I use catheter-directed interventional therapy to treat patients with venous thromboembolism. Blood. (2018) 131(7):733–40. 10.1182/blood-2016-11-693663 29295847 PMC5814931

[11] Goldhaber SZ Magnuson EA Chinnakondepalli KM Cohen DJ Vedantham S. Catheter-directed thrombolysis for deep vein thrombosis: 2021 update. Vasc Med. (2021) 26(6):662–9. 10.1177/1358863X211042930 34606385 PMC9009765

[12] Vedantham S Salter A Lancia S Lewis L Thukral S Kahn SR. Clinical outcomes of a pharmacomechanical catheter-directed venous thrombolysis strategy that included rheolytic thrombectomy in a multicenter randomized trial. J Vasc Intervent Radiol. (2021) 32(9):1296–309.e7. 10.1016/j.jvir.2021.06.001 PMC8818274 34119655

[13] Sailer A Revzin MV Pollak J Ayyagari R Mojibian HR Nezami N Deep vein thrombosis: update on mechanical thrombectomy and intravascular US. RadioGraphics. (2022) 42(6):E184–E5. 10.1148/rg.220031 36190870

[14] Shah AD Bajakian DR Olin JW Lookstein RA. Power-pulse spray thrombectomy for treatment of paget-schroetter syndrome. Am J Roentgen. (2007) 188(5):1215–7. 10.2214/AJR.06.0028 17449762

[15] Yusuf S Whitaker S Gregson R Wenham P Hopkinson B Makin G. Experience with pulse-spray technique in peripheral thrombolysis. Euro J Vasc Endovasc Surg. (1994) 8(3):270–5. 10.1016/S0950-821X(05)80141-0 8013676

[16] Suarez JA Meyerrose GE Phisitkul S Kennedy S Roongsritong C Tsikouris J Review of catheter thrombectomy devices. Cardiology. (2004) 102(1):11–5. 10.1159/000076996 14988612

[17] Kiang S Rigberg D. Surgical and pharmacomechanical venous thrombectomy. In: Mansour MA Mitchell E Shames M, editors. Atlas of Vascular & Endovascular Surgical Techniques. New Delhi, India: Jaypee Brother Medical Publishers (2015). p. 395.

[18] Lichtenberg MK Stahlhoff S Młyńczak K Golicki D Gagne P Razavi MK Endovascular mechanical thrombectomy versus thrombolysis in patients with iliofemoral deep vein thrombosis–a systematic review and meta-analysis. Vasa. (2021) 50(1):59–67. 10.1024/0301-1526/a000875 32449481

[19] Hoexum F Hoebink M Coveliers HM Wisselink W Jongkind V Yeung KK. Management of paget-schroetter syndrome: a systematic review and meta-analysis. Euro J Vasc Endovasc Surg. (2023) 66:866–75. 10.1016/j.ejvs.2023.08.065 37678659

[20] Flumignan RLG Civile VT Areias LL Flumignan CDQ Amorim JE Lopes RD Stenting or angioplasty for the treatment of deep vein thrombosis: systematic review and meta-analysis of randomized controlled trials. Medicine (Baltimore). (2023) 102(22):e33924. 10.1097/MD.0000000000033924 37266612 PMC10237682

[21] Heil J Miesbach W Vogl T Bechstein WO Reinisch A. Deep vein thrombosis of the upper extremity: a systematic review. Dtsch Arztebl Int. (2017) 114(14):244. 10.3238/arztebl.2017.0244 28446351 PMC5415909

[22] Huang Y Abad-Santos M Iyer RS Monroe EJ Malone CD. Imaging to intervention: thoracic outlet syndrome. Clin Imag. (2022) 89:23–36. 10.1016/j.clinimag.2022.06.003 35689965

[23] Khalilzadeh O Glover M Torriani M Gupta R. Imaging assessment of thoracic outlet syndrome. Thorac Surg Clin. (2021) 31(1):19–25. 10.1016/j.thorsurg.2020.09.002 33220768

[24] Kim TI Sarac TP Orion KC. Intravascular ultrasound in venous thoracic outlet syndrome. Ann Vasc Surg. (2019) 54:118–22. 10.1016/j.avsg.2018.08.077 30217714

[25] Schropp L de Kleijn RJ van Hattum ES Petri B-J Vonken E-J de Borst GJ. Intravascular ultrasound in the management of venous thoracic outlet syndrome. Euro J Vasc Endovasc Surg. (2022) 63(1):161–2. 10.1016/j.ejvs.2021.10.042 34824010

[26] Tsekouras N Comerota AJ. Current trends in the treatment of venous thoracic outlet syndrome: a comprehensive review. Intervent Cardiol. (2014) 6(1):103. 10.2217/ica.13.91

[27] Vik A Holme PA Singh K Dorenberg E Nordhus KC Kumar S Catheter-directed thrombolysis for treatment of deep venous thrombosis in the upper extremities. Cardiovasc Intervent Radiol. (2009) 32:980–7. 10.1007/s00270-009-9655-y 19641959

[28] Sharafuddin MJ Sun S Hoballah JJ. Endovascular management of venous thrombotic diseases of the upper torso and extremities. J Vasc Intervent Radiol. (2002) 13(10):975–90. 10.1016/S1051-0443(07)61861-2 12397118

[29] Stuck AK Engelberger RP Saengprakai W Kucher N. Pharmacomechanical or ultrasound-assisted thrombolysis, balloon angioplasty and provisional surgical decompression for upper extremity deep vein thrombosis due to thoracic outlet syndrome. Thromb Res. (2016) 145:109–11. 10.1016/j.thromres.2016.08.006 27543940

[30] de Kleijn RJ Schropp L Westerink J de Borst GJ Petri B-J. Timing of thoracic outlet decompression after thrombolysis for primary upper extremity deep venous thrombosis: a systematic review. Ann Vasc Surg. (2020) 66:654–61. 10.1016/j.avsg.2020.01.083 32035261

[31] Tabriz DM Arslan B. Management of central venous stenosis and occlusion in dialysis patients. Sem Intervent Radiol. (2022) 39(01):051–5. 10.1055/s-0041-1742152 PMC8856783 35210733

[32] Rashwan B Shwaiki O Partovi S Karuppasamy K Gill A Gadani S. Thoracic central venous occlusion from the interventional radiology perspective. Cardiovasc Diag Ther. (2023) 13(1):299–310. 10.21037/cdt-22-93 PMC9971304 36864973

[33] Dadashzadeh ER Ohman JW Kavali PK Henderson KM Goestenkors DM Thompson RW. Venographic classification and long-term surgical treatment outcomes for axillary-subclavian vein thrombosis due to venous thoracic outlet syndrome (paget-schroetter syndrome). J Vasc Surg. (2023) 77(3):879–89.e3. 10.1016/j.jvs.2022.11.053 36442701

[34] Karaolanis G Antonopoulos CN Koutsias SG Giosdekos A Metaxas EK Tzimas P A systematic review and meta-analysis for the management of paget-schroetter syndrome. J Vasc Surg Veno Lymph Dis. (2021) 9(3):801–10.e5. 10.1016/j.jvsv.2021.01.011 33540134

[35] de Kleijn R Schropp L Westerink J Nijkeuter M Jv L Teijink J Current treatment strategies for primary upper extremity deep venous thrombosis; a retrospective observational multicentre case series. Front Surg. (2022) 9:1080584. 10.3389/fsurg.2022.1080584 36620382 PMC9815523

[36] Davies MG Hart JP. Venous thoracic outlet syndrome and hemodialysis. Front Surg. (2023) 10:1149644. 10.3389/fsurg.2023.1149644 37035557 PMC10073697

